# The prediction of the progressive deformation mode based on active waveguide-generated acoustic emission

**DOI:** 10.1038/s41598-026-43457-0

**Published:** 2026-03-10

**Authors:** Zhihui Wu, Yunlong Sun, Jie Dong, Bo Liu, Yongxin Yu, Lingjun Zhang

**Affiliations:** 1 College of Civil Engineering, Hebei University of Architecture, No.13 Chaoyang Road, Zhangjiakou, 075000 Hebei China; 2 Hebei Provincial Key Laboratory of Civil Engineering Diagnosis, Renovation and Disaster Resistance, No.13 Chaoyang Road, Zhangjiakou, 075000 Hebei China

**Keywords:** Landslide, Multiple AE features, Identification reference, Early warning, Engineering, Natural hazards

## Abstract

Buildings on the slope are inevitably affected by the stability of the slope, and cracks and uneven settlement appear in the building structure during the landslide. The prediction of landslides is very important to the judgment of building safety. To investigate the precursor detection of landslide failures based on acoustic emission (AE) signal, a model test aiming at reproducing the shear surface deformation of typical landslide mode was designed. The evolution characteristics of the AE signals were analyzed in terms of AE count, cumulative AE count, AE correlation diagrams, and time-frequency properties. The test results show that for the progressive deformation mode, the AE count experiences a low-level period, an active period and a rapid increase period, and the distribution of the correlation diagram concentrates in a relatively small scale and then gradually scatters. There are high-frequency signals during the accelerating deformation stage. In laboratory experiments, the gray catastrophe analysis model can effectively predict sliding instability states. The comprehensive use of multiple AE features helps to more accurately identify landslide deformation, providing valuable references for subsequent research.

## Introduction

**Fig. 1:**
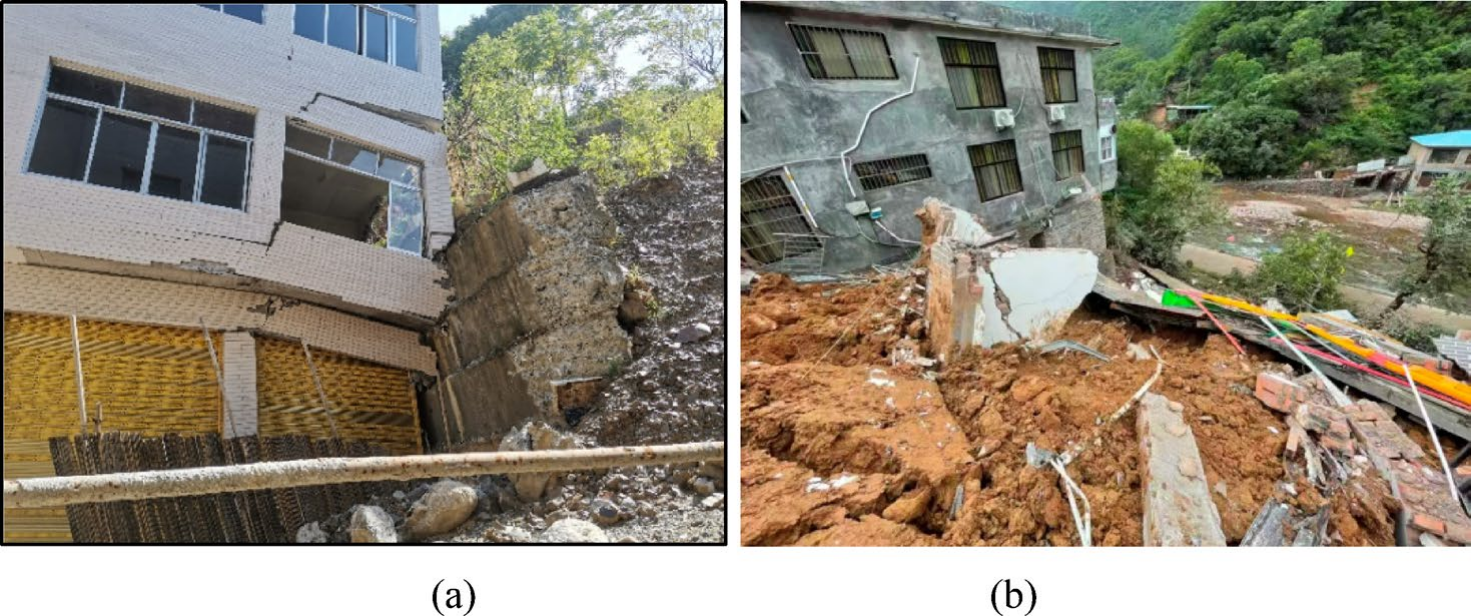
Distribution and destruction of buildings on the slope.

 Due to the development of regional economy and infrastructure, many buildings are built on the slope. In fact, landslide is considered one of the main geological hazards in the world, which has caused enormous loss of life and property^1–4^. Due to different geological conditions and environmental factors, such as rainfall, earthquake and fluctuation of reservoir level, there are a large number of geologic hazards^5–8^, especially the slope damage has a huge threat on mountainous buildings and factory infrastructures, as shown in Fig. 1. At present, there is the greater magnitude of potential landslides in many regions of the world, i.e. more than 5000 landslides or potential landslides have been identified in the Three Gorges Reservoir(TGR) region, which causes the enormous threat from geological hazards^9^. Therefore, it is urgent to improve the identification and warning accuracy of the landslide, which provides the reference for the evaluation of building safety.

Based on the real-time monitoring methods, identifying landslide movement effectively and providing early prediction information are both very important^10–13^. There is an urgent need to understand the deformation characteristics and kinematic evolution process of the landslide, thus landslide failures can be forecasted in a timely and relatively accurate manner. Further, prevention measures will be taken to enable the evacuation of vulnerable people and maintenance of critical buildings, and the losses caused by such natural disasters will be minimized^14–17^.

The various landslides have different deformation modes and failure behaviors. Therefore, monitoring the physico-mechanical parameters during the slope deformation process is very important to judge the damage level and disaster mechanism of the landslide. AE has recently been recognized as a feasible technique to monitor landslide movement, which could contain rich information of the landslide evolution process. For decades, researchers have used the measurement and quantification of AE signals generated by the sliding deformation of soil slopes to develop monitoring technology strategies^18–21^. These methods usually use waveguides to provide a low attenuation propagation path of acoustic emission signals. Later, researchers developed active waveguide devices^22,23^, and abundant AE signals are generated by the backfill material. When the slope is sliding, the soil mass will squeeze the backfill material, causing particle-particle interaction to release AE signals. Subsequently, many landslide tests have shown that the slope displacement rate is directly related to the AE rate generated by the active waveguide. As the deformation rate increases, the AE rate is proportional to the applied displacement rate^24–26^.

Since scholar Saito carried out landslide prediction based on the creep test results, many researchers have been studying and exploring the slope failure behaviors and landslide prediction methods^27–29^. With the increasing number of landslide monitoring cases, the movement patterns of different landslides have been widely recorded and investigated by researchers and geologists all over the world, and the monitoring data shows that there are many types of landslides, mainly including progressive landslide mode^30–32^. For the progressive landslide instability, in the deformation process of the shear sliding surface, the waveguide device with the material column is subjected to different confining pressures, thus the interaction between the particles and the scale of contact stress in the shear zone is significant variation, resulting in difference AE signals emitted. Specifically, the evolution characteristics of AE parameters are critical for the identification and warning of the progressive landslide.

To date, some researchers have been investigating early warning of slope failures. Although numerous monitoring techniques, evaluation criteria, warning patterns are available, and they are currently widely used for monitoring landslide geological hazards, the effective identification reference and warning determination of the landslide are always a difficult problem, which is not conducive to the prevention and control of landslide hazards. So it becomes important to explore effective identification indicators of the landslide failure, further to provide effective evaluation to buildings in the immediate vicinity of the landslide.

In this study, we conducted physical model experiments to simulate the progressive landslide mode by controlling deformation rates. The AE characteristic parameters, such as the AE count, amplitude, duration, dominant frequency, were analyzed. Furthermore, the tests indicate that the AE characteristic parameters demonstrate the significant evolution characteristic with the shear surface deformation, and the comprehensive multi-feature analyzing approach may provide effective solutions for detecting physical activities related to progressive landslide deformation, also give early warning information to such failure.

## Mechanical mechanism of progressive landslide mode

The mechanical mechanism of progressive landslides is mainly driven by the force imbalance on the sliding surface, where the shear stress exceeds the long-term strength of the soil, triggering creep deformation. This process is influenced by external factors such as rainfall, leading to deformation evolving from the initial stage through the stable stage to the accelerated stage, ultimately causing landslide instability. This mechanism is particularly relevant to building safety, manifesting as progressive structural damage (e.g., crack propagation and uneven settlement).

## Experimental system and test procedure

### Experimental apparatus

A laboratory shear apparatus was developed to reproduce the deformation behavior of a soil slope sliding surface. The device consists of two rigid steel containers, each measuring 0.2 × 0.2 × 0.3 m. The lower container was rigidly anchored to a heavy steel base to ensure stability and eliminate unintended movement during testing. Prior to each experiment, a lubricant was applied along the contact interface between the upper and lower containers to minimize interface friction and ensure that the induced deformation was primarily governed by soil shear behavior. Both containers were filled with prepared soil material to represent a representative element of the slope mass. In addition, a steel waveguide could be vertically embedded within the soil column and surrounded by granular backfill material, allowing effective transmission and monitoring of acoustic emission signals generated during deformation.

**Fig. 2:**
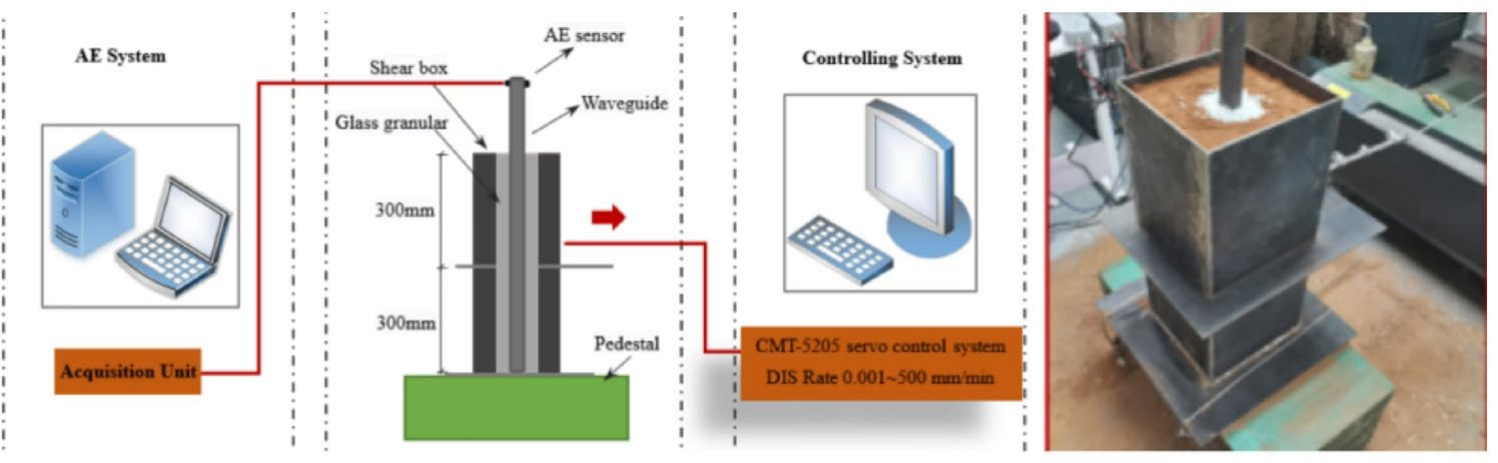
Experimental apparatus.

The loading system employed in the experiments was a CMT-5205 electro-hydraulic servo-controlled testing machine. This system integrates an oil pump and an electro-hydraulic servo valve, enabling automated loading control with precise regulation of displacement variables. The apparatus has a maximum loading capacity of 300 kN, while the displacement rate can be continuously adjusted within a range of 0.001 to 500 mm/min, fully satisfying the requirements of the present experimental program. During testing, a predefined control program was used to maintain a constant displacement rate. The loading device applied tensile force by pulling a steel wire rope, which was horizontally connected to the upper container. This configuration generated controlled shear deformation within the soil column, thereby simulating the displacement evolution of a slope sliding surface. A schematic illustration of the experimental setup and loading mechanism is provided in Fig. 2.

### Soil and backfills material

The 80 mm diameters hole was reserved by the PVC tube matched the size. The waveguide, a 30 mm dia., 1.0 m long steel tube with 5 mm wall thickness was installed in the center of the hole with the columnar backfills. Then the soil element model was prepared using the layered filling method. The filling height of each layer was 15 cm, and the soil was uniformly compacted with a constant external force. and the specific particle parameters are shown in Table 1.

**Table 1:** Physico-mechanical properties of model soil.

Water content (%)	Elasticity modulus (Gpa)	Density (kg/m^3^)	Cohesion (kPa)	Friction angle (°)
17.8	0.12	1800	21.0	24.0

Besides, among the series tests on the waveguide with different backfills (glass sand, marble gravel, river sand), the glass sand backfills case shows remarkable variation characteristics for the cumulative AE count, which is closely related to the sliding deformation process. Besides, there is prior AE detection sensitively when using the glass sand backfills compared to the other cases during the initial stage with an extremely slow deformation rate. Moreover, the quantitative correlational relationship between the cumulative AE count and shear deformation is the potential application for detecting pre-failure processes on soil slope, and providing timely information on the movement status of the progressive landslide. Subsequently, a series of displacement-controlled shear tests were conducted on waveguide models with glass sand backfills, to study the AE detection characteristics under different shear movements, and the specific particle parameters are shown in Table 2.

**Table 2:** The mechanical parameters properties of backfill materials.

Backfills	Size range (mm)	Particle density: (kg/m^3^)	Dry density (kPa)	Void ratio
glass sand	2 ~ 5	2936	1600	0.72

### AE measurement system

The DS2AE equipment was adopted, which can collect and display AE signal waveforms and parameters in real-time. The AE sensor is a Nano-30 resonant high-sensitivity sensor with a frequency range of 125 ~ 750 kHz. The AE sampling threshold value was 50 dB to effectively reduce the noise impact. The sensor was fixed with white tape, and the coupling agent was applied between the sensor and the waveguide to reduce the signal attenuation. During the loading process, the displacement-time information and AE waveforms were collected synchronously.

### Test procedure

**Fig. 3:**
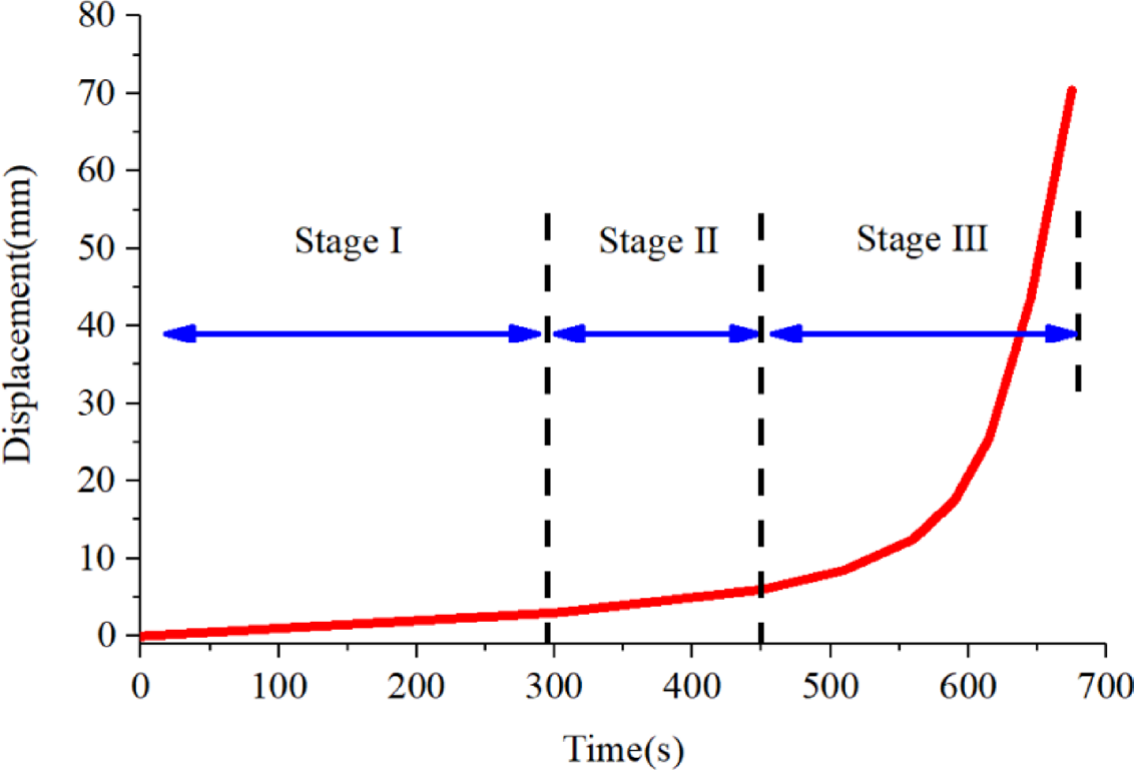
Displacement-time curves of progressive deformation mode.

The displacement-time functions were designed to reproduce the evolution process of the progressive landslide mode, the tests was controlled by displacement rate. Through the control system of the test machine, the wire rope linked to the shear box will respond in time based on the displacement rate parameters, which can accurately control the deformation rate of the shear surface according to the given parameters formulas. The displacement-time curve of the shear sliding surface was automatically controlled, as shown in Fig. 3. Thus, the testing process was stable and a smooth displacement-time curve could be obtained. After all stages of operation, the testing program automatically terminated, and the shear deformation process was completed.

## Results and analysis

### AE count and shear surface deformation

In the progressive deformation mode, the AE count demonstrates distinct evolutionary characteristics corresponding to shear surface displacement. During the initial phase, AE activity remains at a comparatively low level, with minimal acoustic emission signals detected. As deformation progresses to the stable stage, AE counts become notably more active, showing increased frequency of occurrence. The most significant change occurs when shear displacement enters the accelerated stage, where AE signals exhibit abrupt increases, characterized by intense burst characteristics.

**Fig. 4:**
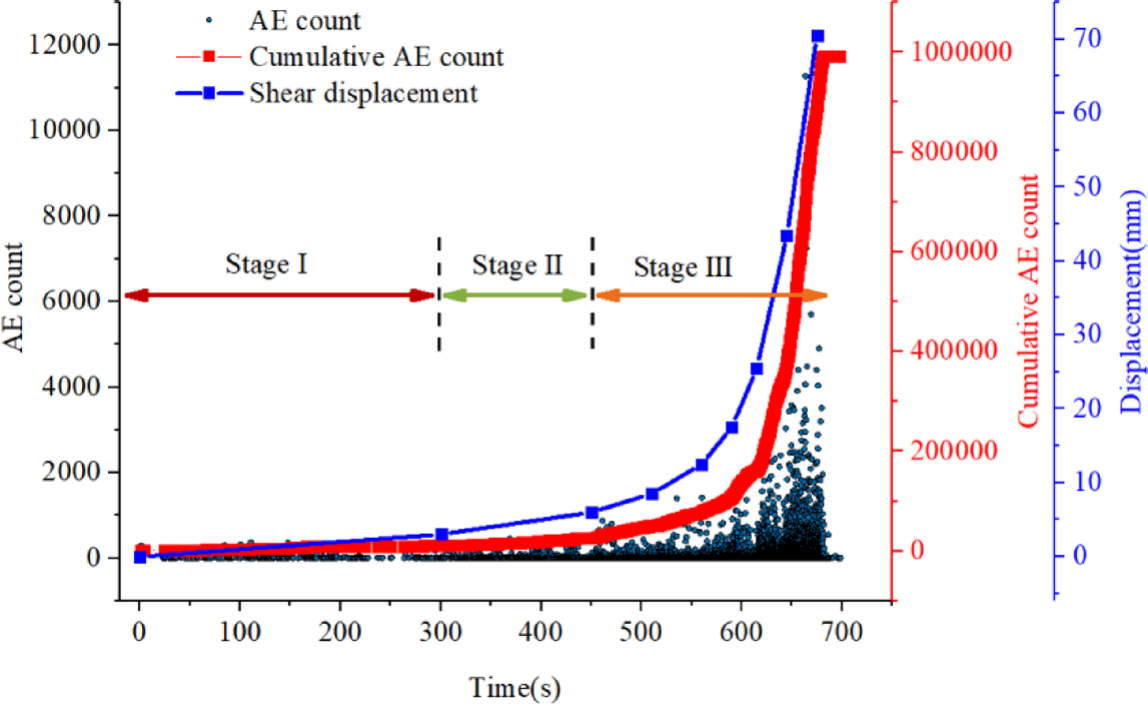
The AE count, cumulative AE count and shear surface displacement for progressive deformation mode.

Notably, As shown in Fig. 4, the cumulative AE count curves display remarkable consistency with the shear surface displacement pattern. Under low deformation rates, the cumulative AE count increases gradually, while during the accelerated phase, both parameters show synchronous rapid growth, confirming their strong correlation in progressive landslide modes.

**Fig. 5:**
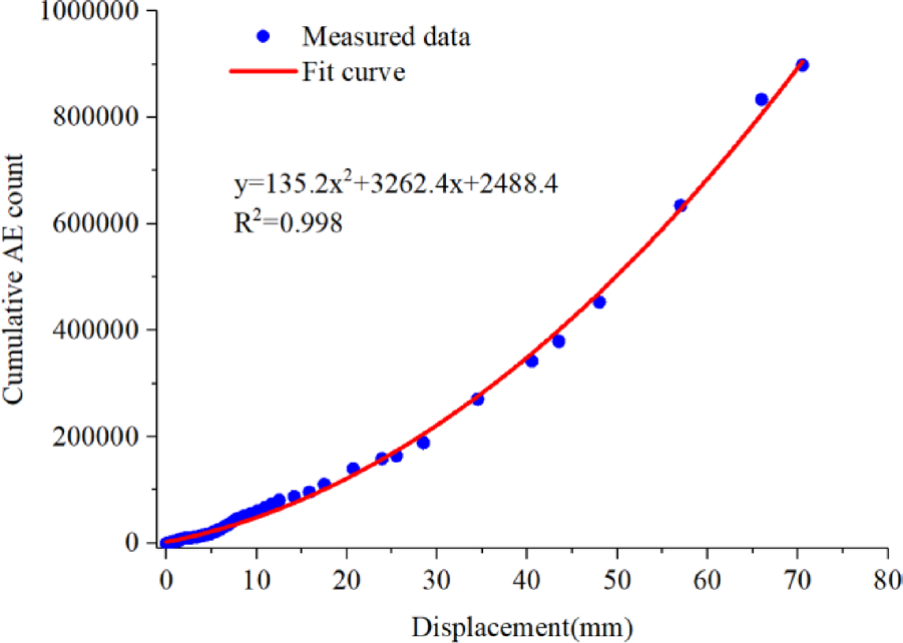
Cumulative acoustic emission count vs. displacement.

As shown in Fig. 5, the cumulative AE counts exhibit a clear nonlinear increasing trend with increasing displacement. During the initial deformation stage, the cumulative AE counts increase slowly. With further displacement, especially upon entering the accelerated deformation stage, AE events become increasingly concentrated, leading to a significant increase in the growth rate of cumulative AE counts. The relationship between cumulative AE counts and displacement can be well described by a quadratic function, indicating a strong consistency in their evolutionary behavior.

These results indicate that the accumulation of acoustic emission activity is closely associated with the overall deformation evolution of the specimen. The cumulative AE counts can effectively characterize the progressive development of internal damage during the shear deformation process.

### Evolution characteristics of AE duration-count

As an analysis method of AE events, the correlation analysis method can demonstrate the correlation characteristics of different AE signals. In this section, the correlation diagram of AE duration-count is used to analyze the signal evolution process under the progressive mode. The emitted AE signal may carry different waveform parameters under different deformation rates.

**Fig. 6:**
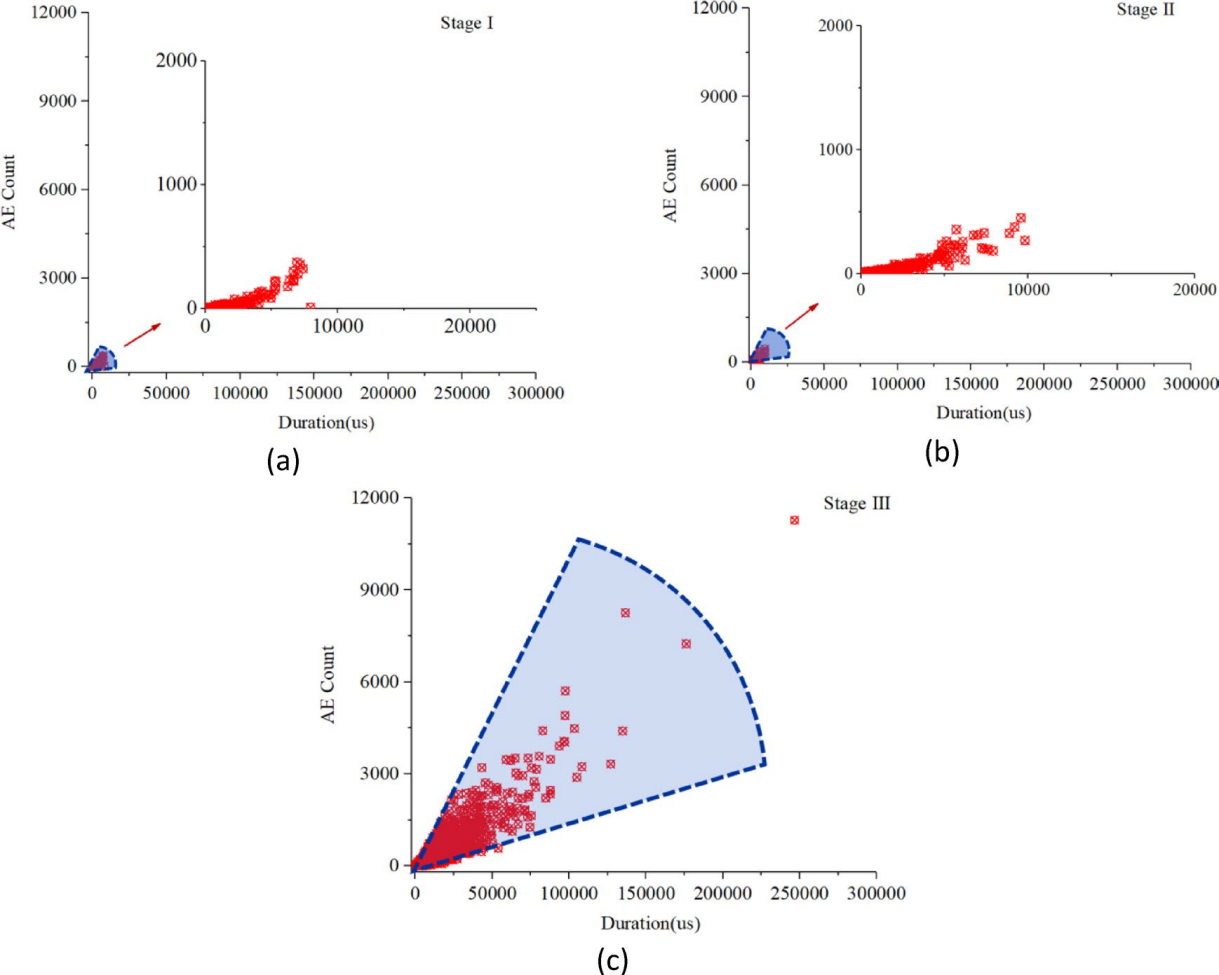
Evolution process of AE duration-count under the progressive deformation modes.

Figure 6 presents the correlation diagram of AE duration-count in the progressive deformation mode. In the initial deformation stage, the AE activity is not active, and the AE signal duration and AE count are both at a low level, whereas the signal distribution concentrates in a relatively smaller scale. As the deformation begins to enter the accelerating stage gradually, the AE signals are accompanied by a longer duration, and the magnitude of the AE count increases simultaneously. There is a noteworthy phenomenon that the AE duration-count shows a positive correlation on a large scale, and the signal distribution is relatively scattered close to the later stage.

### Evolution characteristics of AE amplitude-energy

**Fig. 7:**
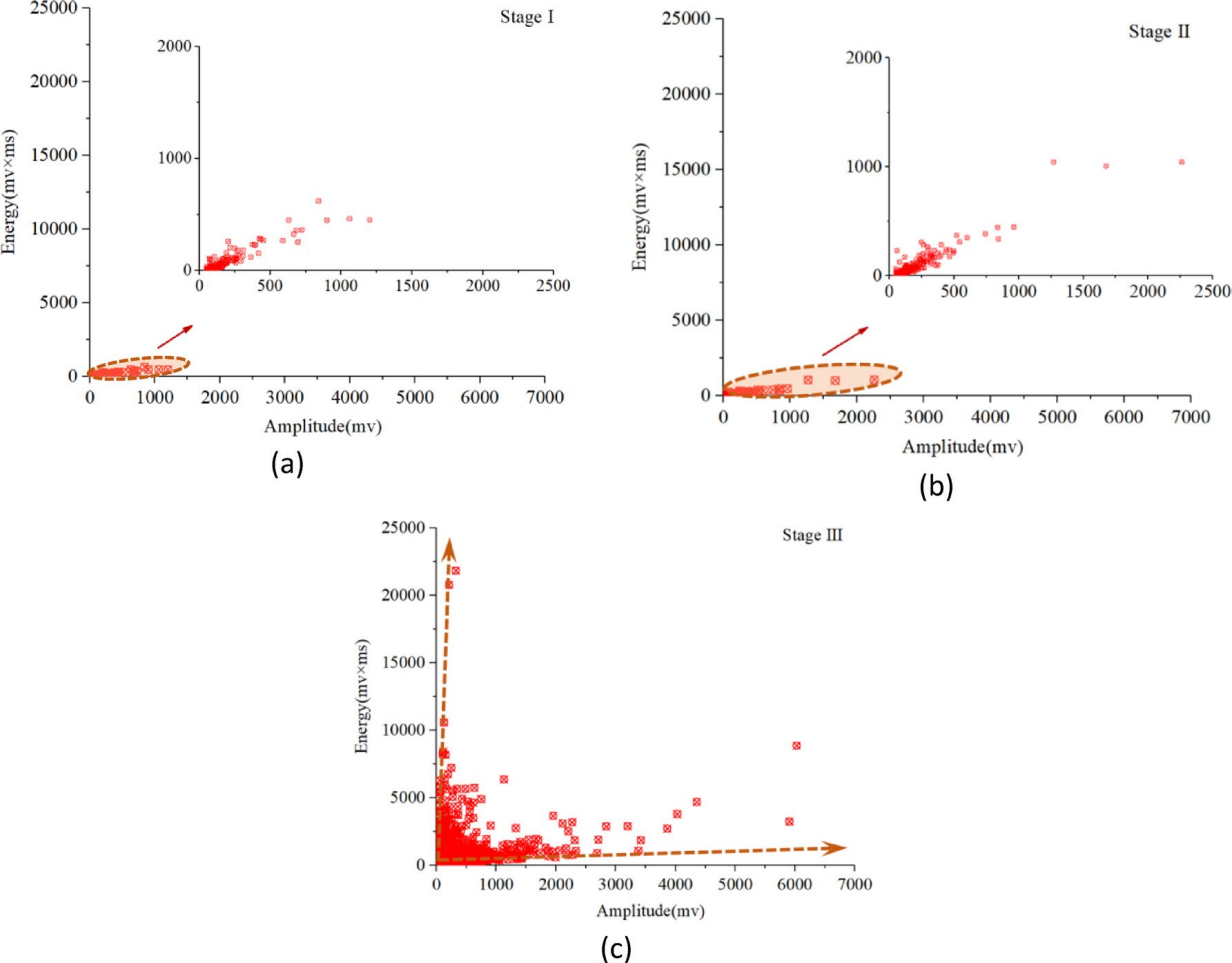
Evolution process of AE amplitude-energy under the progressive deformation mode.

Figure 7 presents the correlation diagram of AE amplitude-energy in the progressive deformation mode. In the initial stage, the AE activity maintains at a low level, the signal amplitude and energy are both at a relatively low level, and the signal hits distribution concentrates in a relatively small scale. As the deformation rate increase, the AE signal has a larger magnitude of AE amplitude and energy. Relatively, the amplitude-energy distribution range begins to gradually expand widely, with a large number of high-amplitude and low-energy, low-amplitude and high-energy AE signals, and there is a noteworthy phenomenon that the signal hits distribution has relatively scattered during the final stage.

### Dominant frequency characteristic

During the process of shear surface deformation, the reaction from the host soil causes the pressure along columnar backfills changes constantly, resulting in the confining pressures on the backfill fluctuation, this behavior affects the frequency domain characteristics of the AE signal directly^33–36^. For the glass particle shearing movement, such AE probably carries crucial information concerning the variant landslide process. Thus the frequency domain characteristics may present distinction properties during the progressive deformation, further provide a basis for the prediction of the landslide.

**Fig. 8:**
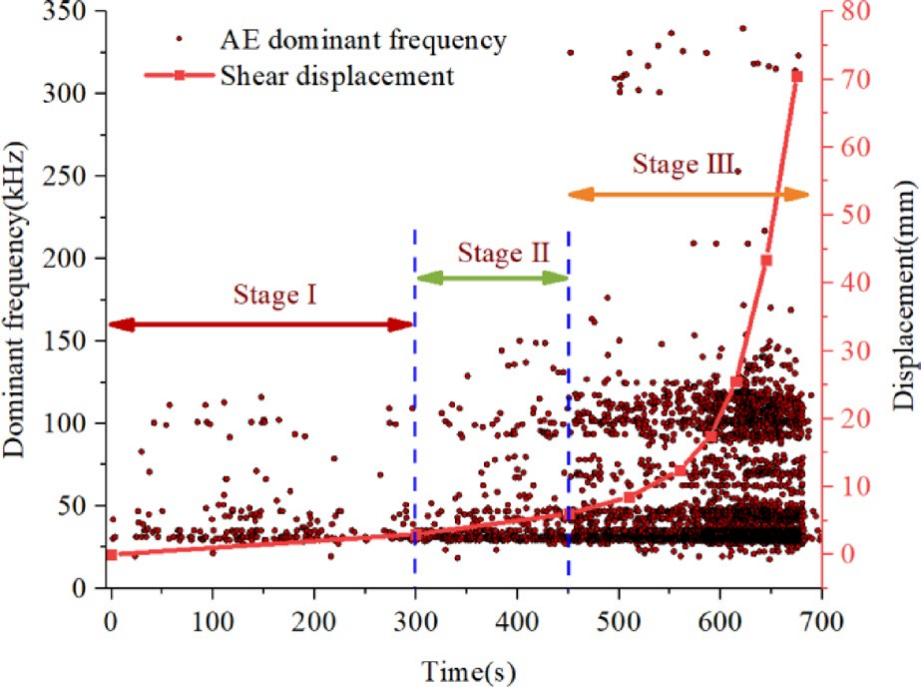
The AE dominant frequency evolution and shear displacement in the entire test process.

As can be seen from Fig. 8, The spectral characteristics of AE signals show clear variations across different stages of shear deformation. AE signals with frequencies below 150 kHz are classified as low-frequency components and are predominantly observed during the initial stage of the experiment. These signals are mainly associated with minor frictional interactions among the internal components of the waveguide system. With an increase in the shear deformation rate of the soil, the number of AE events gradually increases, indicating intensified internal structural adjustments and enhanced contact interactions.

The simultaneous occurrence of high-frequency signals in the range of 300–350 kHz together with densely distributed low- and mid-frequency signals is typically observed under conditions of continuously increasing confining pressure. This spectral pattern corresponds to changes in the interparticle contact state within the soil, including enhanced interparticle friction and an accelerated compaction process. It should be emphasized that the amplification of signals in this high-frequency band cannot be attributed solely to the micro-mechanical behavior of the soil. The frequency response characteristics of the AE sensors and the transmission effects of the waveguide system also play important roles.Due to the higher sensitivity of the sensors within specific frequency ranges, high-frequency components may be preferentially amplified. Moreover, frequency-dependent attenuation and dispersion induced by waveguide propagation can further influence the recorded spectral distribution. Under these considerations, part of the high-frequency AE signals is likely associated with energy release during particle breakage.

### Grey cusp catastrophe model

Combining the grey system theory and the catastrophe theory analysis method, a practical grey-catastrophe prediction model based on the AE count parameter sequence is constructed, thereby realizing accurate prediction and determination of potential instability during the loading test of waveguide structures.

During the process of acoustic emission monitoring, to reduce the randomness of the time series of AE count parameters and enhance the utilization of useful information in the sequence, the grey theory can be applied to process the original acoustic emission data, thereby weakening its randomness. The AE count parameter is a time-ordered data series. By establishing the grey GM(1,1) model, prediction research can be carried out. The GM(1,1) model is based on the random original AE count parameter sequence, and a new predicted AE count sequence is formed through accumulation.

Basic Principle of the GM(1,1) Prediction Model: Let ${x^{(0)}}=({x^{(0)}}(1),{x^{(0)}}(2), \cdots ,{x^{(0)}}(n))$be the original data sequence, The accumulated generating operation (AGO) sequence is defined as${x^{(1)}}=({x^{(1)}}(1),{x^{(1)}}(2), \cdots ,{x^{(1)}}(n))$, where${x^{(1)}}(k)=\sum\limits_{{i=1}}^{k} {{x^{(0)}}(i)} ,k=1,2, \cdots ,n$, The grey derivative for the sequence is defined as:

1 $$d(k)={x^{(0)}}(k)={x^{(1)}}(k) - {x^{(1)}}(k - 1)$$

The grey differential equation model for GM(1,1) is given by:

2 $$d(k)+a{z^{(1)}}(k)=b$$

For the grey differential equation of GM(1,1), if the moment $k=2,3, \cdots ,n$ of the grey derivative ${x^{(0)}}(k)$is regarded as a continuous variable *t*, and ${x^{(1)}}(t)$ is treated as a function of time *t*,then ${x^{(0)}}(k)$corresponds to the derivative $\frac{{d{x^{(1)}}(t)}}{{dt}}$, and the background value ${z^{(1)}}(k)$ corresponds to ${x^{(1)}}(t)$.Accordingly, the white differential equation corresponding to the grey differential equation of GM(1,1) is as follows:

3 $$\frac{{d{x^{(1)}}(t)}}{{dt}}+a{x^{(1)}}(t)=b$$

As stated above, the following holds:

1. The solution to the whitening equation$\frac{{d{x^{(1)}}}}{{dt}}+a{x^{(1)}}=b$,also known as the time response function, is
  4 $${\hat {x}^{(1)}}(t)=({x^{(1)}}(0) - \frac{b}{a}){e^{ - a(t - 1)}}+\frac{b}{a}$$
2. The time response sequence of the GM(1,1) grey differential equation ${x^{(0)}}(k)+a{z^{(1)}}(k)=b$is
  5 $${\hat {x}^{(1)}}(k+1)=({x^{(1)}}(0) - \frac{b}{a}){e^{ - a(k)}}+\frac{b}{a},k=1,2,...,n$$
3. Setting${x^{(1)}}(0)={x^{(0)}}(1)$,we obtain
  6 $${\hat {x}^{(1)}}(k+1)=({x^{(0)}}(1) - \frac{b}{a}){e^{ - a(k)}}+\frac{b}{a},k=1,2,...,n$$
4. The restored value
  7 $${\hat {x}^{(0)}}(k+1)={\hat {x}^{(1)}}(k+1) - {\hat {x}^{(1)}}(k)$$

Equation (7) serves as the prediction equation for the acoustic emission data series, yielding the predicted values for the acoustic emission data.

Subsequently, analysis can be conducted based on the cusp catastrophe model. Based on the acoustic emission parameter sequence processed by the grey system theory, a power series polynomial is obtained through least squares curve fitting. After differentiating the fitted polynomial, the functional expression of the grey model for acoustic emission parameters is derived as follows:

8 $$y(t)={a_0}+{a_1}t+{a_2}{t^2}+{a_3}{t^3}+{a_4}{t^4}$$

Let, $t=p - q,q={a_3}/4{a_4}$, then Eq. (1) is transformed into the general form of the cusp catastrophe model:

9 $$y(t)={b_0}+{b_1}p+{b_2}{p^2}+{b_4}{p^4}$$

In the equation,

10 $$\left[ {\begin{array}{*{20}{c}} {{b_0}} \\ {{b_1}} \\ {{b_2}} \\ {{b_4}} \end{array}} \right]=\left[ {\begin{array}{*{20}{c}} {{q^4}}&{ - {q^3}}&{{q^2}}&{ - q}&1 \\ { - 4{q^3}}&{3{q^2}}&{ - 2q}&1&0 \\ {6{q^2}}&{ - 3q}&1&0&0 \\ 1&0&0&0&0 \end{array}} \right]\left[ {\begin{array}{*{20}{c}} {{a_4}} \\ {{a_3}} \\ {{a_2}} \\ {{a_1}} \\ {{a_0}} \end{array}} \right]$$

For the convenience of calculation, Eq. (2) is further converted into the standard form of the cusp catastrophe model:

11 $$v(p)={p^4}+u{p^2}+vp+C$$

In the equation, for the specific evolution process of acoustic emission parameters: p represents the acoustic emission parameters of the entire system, indicating the time parameter of the state variable; u and v can represent the control variables of the time-fitted sequence of acoustic emission parameters during the entire monitoring process; the constant term C is usually not considered. The calculation formulas for parameters p, u, and v are as follows:

If $${b_4}>0$$, then:

12 $$p=\left\{ {(t+q)\sqrt[4]{{{b_4}}}} \right\}, u={b_2}/\sqrt {{b_4}}, v={b_1}/\sqrt[4]{{{b_4}}}$$

If $${b_4}<0$$, then;

13 $$p=\left\{ {(t+q)\sqrt[4]{{{\mathrm{-}}{b_4}}}} \right\}, u={\mathrm{-}}{b_2}/\sqrt {{\mathrm{-}}{b_4}}, v={\mathrm{-}}{b_1}/\sqrt[4]{{{\mathrm{-}}{b_4}}}$$

According to the catastrophe theory model, the equilibrium surface equation of the potential function *V(p)* is as follows:

14 $$4{p^3}+2up+v=0$$

The bifurcation set equation is as follows:

15 $$\Delta {\mathrm{=}}8{u^3}+27{v^2}$$

Based on the fundamental properties of catastrophe models, the system state exhibits distinct dynamical behaviors depending on the discriminant value (Δ):

When Δ < 0: The system undergoes abrupt destabilization, transitioning discontinuously between stable equilibria due to bifurcation effects.

When Δ > 0: The system resides in a stable equilibrium, where small perturbations do not induce state changes.

When Δ = 0: The system reaches a critical equilibrium, poised at the bifurcation threshold where minor disturbances may trigger sudden phase transitions.

Based on the loading test of the gradual sliding deformation mode, a mathematical model for the activity characteristics of cumulative AE count was established. This model is used to predict the cumulative AE count sequence during the sliding deformation process, and the grey-catastrophe model is applied to conduct predictive analysis on its catastrophe state. The cumulative AE count data sequence monitored during the sliding deformation process was subjected to grey processing, and the predicted sequence of cumulative AE count data was obtained. Among them, the predicted sequence from 480 s to 620 s is shown in Table 3.

**Table 3:** Grey forecasting data of the cumulative AE count.

Number	Monitoring time(s)	Cumulative AE count	Predicted value	Prediction error(%)
1	480	39,531	34,184	13.52
2	500	47,177	42,549	9.80
3	520	54,520	52,960	2.85
4	540	66,750	65,919	1.24
5	560	78,726	82,049	4.22
6	580	95,766	102,125	6.64
7	600	139,224	127,114	8.69
8	620	183,069	158,217	13.57

The cumulative AE count data obtained from the grey prediction was substituted into the calculation data sequence for least squares fitting, and the corresponding fitting polynomial was obtained after processing. Subsequently, the cusp catastrophe theory was applied to conduct catastrophe analysis on the fitting polynomial of the data sequence. Among them, the catastrophe analysis from 580 s to 620 s is shown in Table 4.

**Table 4:** Catastrophe analysis of the cumulative AE count.

Number	Monitoring time(s)	Cumulative AE count	Predicted value		*u*	*v*	$\Delta$
1	580	95,766	102,125		−24.34	−478.02	6054107.46
2	600	139,224	127,114		2099.35	121592.69	4.73E + 11
3	620	183,069	158,217		−151.47	294.97	−25452584.7

The grey-catastrophe analysis model indicates that the sliding deformation movement will undergo catastrophic instability at 620 s. In the preliminary stage leading to catastrophic instability, under the influence of slow sliding deformation, the development trend of the cumulative AE count-time curve remains relatively gentle, and the sliding deformation is in a stable developmental state. As the displacement rate gradually increases, the extrusion and friction between particles intensify progressively; however, the activity level of acoustic emission events remains non-intensive, and the entire sliding deformation system does not yet exhibit signs of catastrophe. Upon entering the accelerated deformation stage, the deformation rate increases rapidly, the cumulative AE count curve begins to show a steeply increasing trend, the prediction model displays catastrophic characteristics, and the entire sliding deformation system begins to reach an unstable state. The model confirms that catastrophic instability occurs at 620 s, a point where the sliding deformation is in the accelerating stage with a rapidly increasing deformation rate, approaching an unstable state. This prediction shows good agreement with experimental observations, demonstrating the effectiveness of the grey-catastrophe model for predicting sliding deformation instability.

## Discussion

A series of tests have been performed under the progressive deformation mode. This study mainly focuses on the AE detection evolution response to the different displacement-time relations of the progressive deformation mode. Some key monitoring parameters of the AE signals, such as the AE count, cumulative AE count, amplitude, frequency domain, were obtained to investigate the corresponding relationship between the AE detection evolution and shear deformation parameters. It’s found that there is a strong correlational relationship between shear deformation with the cumulative AE count, which provides that the curve of cumulative AE count is effectively indicative of sliding displacement, most previous research about the early landslide warning all investigate the correlational relationship between the AE rate and the sliding displacement^21,24,25^.

The test results show that the evolution characteristics of AE signals are different in response to the applied shear deformation stages. For the progressive mode, the low deformation rate maintains the greater time in the early stages, and the smaller AE count continues for a long time until the accelerating deformation stage, and the number of AE count starts to increase rapidly. In addition, for the signal distribution characteristic of AE duration-count and amplitude-energy correlation, the AE distribution presents the evolution process from the small scale to the scattered distribution in large scale. The evolutionary transformation tendency from the small range to the large range, indicates that landslide movement is undergoing different deformation states. These results demonstrate that signal points distribution of AE duration-count and amplitude-energy appears to be correlated with the deformation movement states, this information can provide identification information and early detection of deformation evolution behavior.

**Fig. 9:**
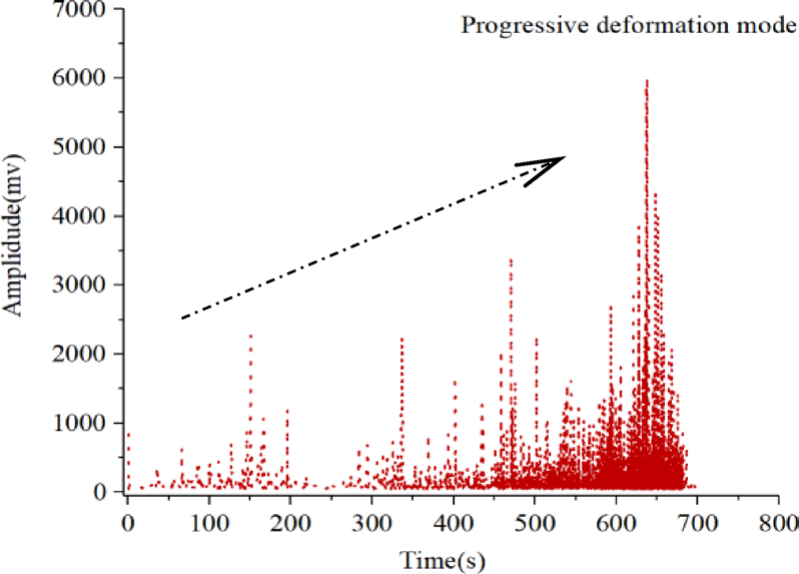
Evolution characteristics of AE amplitude.

**Fig. 10:**
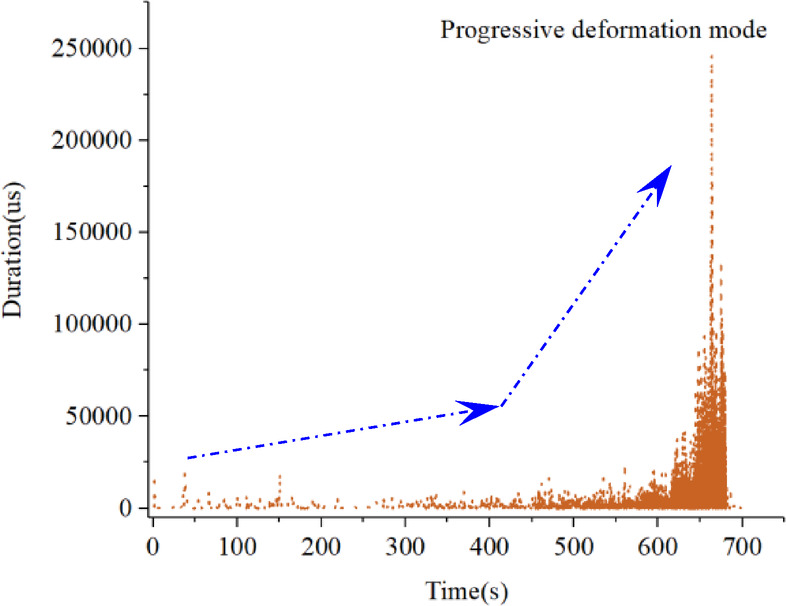
Evolution characteristics of AE duration.

Besides, Figs. 9 and 10 focuses on the evolution process of the AE detection amplitude and duration. The AE amplitude and AE duration are integrated as an identification indicator to understand the overall deformation behavior of slope movements, which reveals relevant evolution characteristics.

It is clear that both the AE detection amplitude and duration experience a low-level period, an active period and a rapid increase period in progressive deformation mode. These AE detection parameters increase to a relatively larger magnitude during the monitoring process, revealing that the landslide deformation rate is getting larger, probably leading to the soil slope entering a dangerous period.

Overall, few studies have focused on the frequency domain characteristic relevant to the deformation evolution stages of landslide failures. By analyzing the evolution characteristics of the frequency domain during different deformation stages, it demonstrates that the frequency domain characteristic is a good discriminant indicator identifying the deformation stage, which has a distinct perception characteristic.

According to the frequency domain indicator of the AE detection signals, which is closely related to the shear deformation stages, especially in the accelerating deformation stage, it may directly determine whether the soil slope is during the rapid deformation stage. The frequency domain has the promising potential to become an efficient warning indicator for such failures. For instance, during the shear deformation stage with rapid deformation rate, not only do the low-frequency and intermediate-frequency signals appear more drastically, also the continual appearance of high-frequency signals between 300 ~ 350 kHz. These high-frequency signals in the accelerating stage are quite distinct from that in the low deformation rate stage. When the shear deformation rate appears in a larger magnitude, note that there is a wide frequency band with high-frequency signal hits.

In summary, different AE detection indexes can be utilized for landslides movement identification and monitoring, in this study, the AE count, cumulative AE count, duration-count scattergram, amplitude-energy scattergram, dominant frequency characteristic overall process are explored to identify the evolution process of the progressive deformation mode. Specifically, we summarize the evolution characteristics of AE detection parameters during different deformation modes, as shown in Table 5.

**Table 5:** Evolution characteristic of AE detection parameters.

AE detection evolution characteristic	Progressive deformation mode
AE count rate	Low-level→ high-level gradually
Curve of the cumulative AE count	Gently increasing→slowly increasing→ sharply increasing
AE duration-count characteristics	Small scale →Large scale gradually
AE amplitude-energy characteristics	Concentration→scatter gradually
Dominant frequency characteristic	Low-frequency→ high-frequency
AE signal amplitude	Low-level→ high-level gradually
AE signal duration	Low-level→ high-level gradually

For the general slope with loose soil or gravel soil subject to continuous long-term rainfall, if the typical AE parameters, such as the count rate, amplitude, duration, present the trend from low level to high level gradually in a long time, and the correlation duration-count and amplitude-energy scattergrams both extend to large scale gradually, notably when the relatively high-frequency signals appear constantly, The slope is in the stage of accelerated deformation.Additionally, if the grey-catastrophe analysis model exhibits a catastrophic phenomenon, it indicates that the slope deformation rate is in a stage of rapid growth and is approaching an unstable state.the progressive landslide instability will possibly occur.

Considering the greater geological complexity of natural slopes, further investigation and field validation are required to provide guidance for future studies. Based on active waveguide–based AE monitoring technology, this study systematically investigated the evolutionary characteristics and precursor patterns of AE signal parameters through laboratory-scale physical model tests and theoretical analyses.

To enhance the reliability of waveguide AE technology for slope geohazard monitoring and early warning, additional research is necessary. Future work should focus on experimentally determining key parameters of the waveguide AE monitoring system, including waveguide rod dimensions, borehole size, and acquisition threshold settings. Furthermore, in situ destructive tests on natural slopes should be conducted to calibrate AE parameters through regression analysis, thereby establishing reliable quantitative indicators or empirical relationships. These efforts will facilitate the more effective application of waveguide-based AE monitoring technology in practical slope engineering.

## Conclusions

In this paper, a model test for reproducing the typical progressive deformation was designed, the displacement, AE data, AE correlation diagram, and frequency domain characteristics were obtained through the experiment. The primary conclusions are drawn:

1. The curve of cumulative AE count shows a steeply upward trend when the deformation increases sharply in the accelerating stage. For the progressive deformation mode, the AE count experiences a low-level period, an active period and a rapid increase period gradually. The correlation distribution of the AE duration-count and amplitude-energy concentrated in a relatively small scale in the initial stage with a low deformation rate. The signal distribution becomes relatively scattered and expands into a large scale as the deformation rate increases.
2. The AE detection during the different deformation stages exhibits evolution characteristic as for the dominant frequency domain. Under the rapid deformation rate, not only the number of the low-frequency and intermediate-frequency signals increase drastically, while the continuous high-frequency signals also increase significantly. For the progressive deformation mode, the frequency domain presents low-frequency signals first then the additional high-frequency signals.
3. By analyzing the AE count sequence using the grey-catastrophe analysis model, it is found that when the model exhibits a catastrophic phenomenon, the sliding deformation is in the acceleration stage, the deformation rate is in the stage of rapid growth, and the sliding deformation is approaching an unstable state. This indicates that the grey-catastrophe model has a good effect on predicting the instability of sliding deformation.
4. It should be noted that the experimental conditions in the laboratory are relatively idealized and therefore differ to some extent from those of real slopes. The scale of the experimental model is limited, and the rock–soil materials are relatively homogeneous, which makes it difficult to fully capture the pronounced scale effects and structural heterogeneity commonly present in natural slopes. As a result, deviations may exist in the propagation and attenuation characteristics of AE signals.

In addition, the noise level in laboratory environments is generally low, whereas field monitoring is susceptible to various sources of environmental noise, such as rainfall, wind loading, traffic-induced vibrations, and construction disturbances, which can significantly increase the difficulty of effective signal identification. Moreover, the installation conditions and construction constraints of waveguide structures in the field—including layout configuration, coupling quality, and long-term stability—are difficult to fully replicate under laboratory conditions.

Therefore, the conclusions of this study are primarily intended to elucidate the AE response mechanisms and the evolutionary characteristics of early warning indicators. Their engineering applicability still requires further validation through long-term field monitoring.

## Data Availability

The raw data can be obtained from the corresponding author (Wuzhihui199245@hotmail.com) upon reasonable request.
